# TDP-43 misexpression causes defects in dendritic growth

**DOI:** 10.1038/s41598-017-15914-4

**Published:** 2017-11-15

**Authors:** Josiah J. Herzog, Mugdha Deshpande, Leah Shapiro, Avital A. Rodal, Suzanne Paradis

**Affiliations:** Department of Biology, Volen Center for Complex Systems, and National Center for Behavioral Genomics, Brandeis University, Waltham, Massachusetts 02454 USA

## Abstract

Amyotrophic Lateral Sclerosis (ALS) and Frontotemporal Dementia (FTD) share overlapping genetic causes and disease symptoms, and are linked neuropathologically by the RNA binding protein TDP-43 (TAR DNA binding protein-43 kDa). TDP-43 regulates RNA metabolism, trafficking, and localization of thousands of target genes. However, the cellular and molecular mechanisms by which dysfunction of TDP-43 contributes to disease pathogenesis and progression remain unclear. Severe changes in the structure of neuronal dendritic arbors disrupt proper circuit connectivity, which in turn could contribute to neurodegenerative disease. Although aberrant dendritic morphology has been reported in non-TDP-43 mouse models of ALS and in human ALS patients, this phenotype is largely unexplored with regards to TDP-43. Here we have employed a primary rodent neuronal culture model to study the cellular effects of TDP-43 dysfunction in hippocampal and cortical neurons. We show that manipulation of TDP-43 expression levels causes significant defects in dendritic branching and outgrowth, without an immediate effect on cell viability. The effect on dendritic morphology is dependent on the RNA-binding ability of TDP-43. Thus, this model system will be useful in identifying pathways downstream of TDP-43 that mediate dendritic arborization, which may provide potential new avenues for therapeutic intervention in ALS/FTD.

## Introduction

Amyotrophic Lateral Sclerosis (ALS) and Frontotemporal Dementia (FTD) are clinically linked, rapidly progressing neurodegenerative diseases that affect both distinct and overlapping regions of the brain. Nearly 50% of ALS patients show some level of cognitive impairment while 25–30% of FTD patients exhibit motor neuron dysfunction^1–4^. Mutations in the TARDBP gene which encodes TAR DNA-binding protein of 43 kDa (TDP-43) have been linked to both ALS and FTD in humans^5,6^. The connection between ALS and FTD has been further confirmed at the molecular level by the identification of TDP-43 as the major component of ubiquitin–positive inclusions in both ALS and the most common pathological form of FTD^7,8^.

While aggregate-forming proteins are often thought to cause disease via toxic gain-of-function (GOF) mechanisms^9–12^, several lines of evidence suggest that pathologically altered TDP-43 causes neurodegeneration by both GOF and loss-of-function (LOF) mechanisms. First, in patients, cytoplasmic TDP-43 inclusions occur concomitantly with depletion of TDP-43 protein from the nucleus^8^. Second, TDP-43 has been shown to downregulate the expression of its own mRNA transcript^13^, and in transgenic mice, overexpression of human TDP-43 results in downregulation of endogenous TDP-43^14^, suggesting an impairment of TDP-43 function by an interplay between both LOF and GOF mechanisms. Third, TDP-43 LOF in animal and cellular models phenocopies many defects seen when overexpressing either wild-type or mutant forms of TDP-43 that are implicated in human disease^15–17^. Thus, LOF and GOF hypotheses are not mutually exclusive and it is possible that both mechanisms contribute to neurodegeneration^18^.

TDP-43 is a highly conserved, ubiquitously expressed, multifunctional nucleic acid-binding protein composed of two RNA recognition motifs (RRM), nuclear localization (NLS) and export signals (NES), and a carboxy-terminal glycine rich region. TDP-43 regulates transcription^19,20^, as well as miRNA processing^21–25^ and mRNA splicing^26–32^. Although it mostly resides in the nucleus, TDP-43 also shuttles to the cytoplasm^33^ where it is involved in stress granule formation^34–40^, axonal transport of target mRNAs^41,42^, and mRNA translation^36,43–47^. TDP-43 interacts with several proteins that are implicated in its RNA-processing functions^36,48^ including the heterogenous nuclear ribonucleoprotein complex which binds to the TDP-43 carboxy-terminal domain^49^. Despite the numerous, well-described functions and interactions of TDP-43, it is not well understood exactly which TDP-43-dependent cellular processes become defective in ALS/FTD and contribute to disease etiology.

Defects in dendritic arbor elaboration and maintenance have been associated with many neurodegenerative diseases^50–52^. In animal models of ALS, cortical neuronal degeneration along with regression of apical dendrites has been observed in presymptomatic SOD1^G39A^ mice^53,54^. Cortico-spinal Motor Neurons (CSMNs; known as Betz cells in humans) also show apical dendrite regression in sporadic, familial, and FTD-ALS patients, indicating the loss of the dendritic arbor as an important marker of cellular pathology in ALS^55^. Given that TDP-43 is involved in the splicing, transport, and stability of several mRNAs which encode proteins that play important roles in neuronal arbor elaboration and synaptic function^13,41,56,57^, we asked whether TDP-43 dysfunction altered neuronal morphology.

To explore this question, we took the approach of overexpressing or knocking down TDP-43 in cultured mammalian neurons isolated from rat hippocampus or cortex and asked if manipulating TDP-43 protein levels interfered with dendritic complexity. Our results demonstrate that both overexpression and knockdown of TDP-43 result in reduced dendritic branching, without affecting neuronal viability. In addition, the effect of TDP-43 overexpression on dendritic loss is dependent on TDP-43 RNA binding. Our findings demonstrate that aberrant levels of TDP-43 disrupt neuronal morphology, an observation that has important implications for understanding disease pathology.

## Results

### Increased TDP-43 expression diminishes dendritic complexity

**Figure 1:**
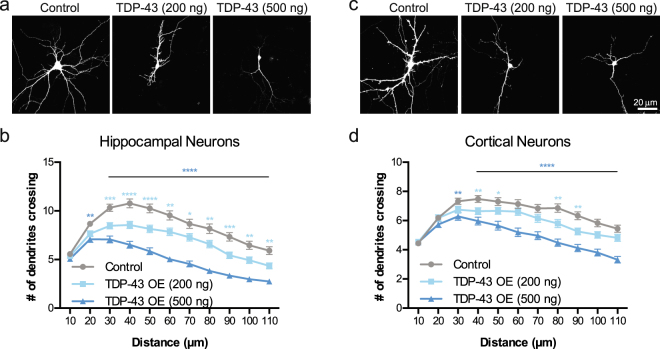
Overexpression of TDP-43 leads to reduced dendritic complexity. Representative images showing DIV 7 (**a**) hippocampal neurons and (**c**) cortical neurons transfected at DIV 2 with a plasmid expressing GFP and an empty vector control, TDP-43 (200 ng/well), or TDP-43 (500 ng/well). Sholl analysis for indicated conditions in (**b**) hippocampal neurons (Control, N = 97; TDP-43 overexpression (200 ng/well), N = 100; TDP-43 overexpression (500 ng/well, N = 80) and **(d)** cortical neurons (N = 90 for each condition). Two-Way ANOVA with Tukey’s Test, *p < 0.05, **p < 0.01, ***p < 0.001, ****p < 0.0001. The colour of the *represents the experimental condition being tested against control. For **(b)**, statistical differences between TDP-43 200 ng/well and TDP-43 500 ng/well are as follows: *at 30 µm, **at 110 µm, ***at 40, 90, and 100 µm, and ****at 50, 60, 70, and 80 µm. For **(d)**, statistical differences between TDP-43 200 ng/well and TDP-43 500 ng/well are as follows: **at 50, 70, 90, and 100 µm, ***at 60 and 80 µm, and ****at 110 µm.

We investigated the effects of TDP-43 overexpression on neuronal morphology in hippocampal and cortical neurons, both of which are susceptible to neurodegeneration in ALS and FTD^58–62^. Our first experiments were performed using cultured prenatal rodent hippocampal neurons. We chose this primary neuronal culture system since it has been used extensively to investigate the cell biological mechanisms of dendritic growth and branching^63–67^ and because of its established use as a model to study TDP-43 biology^68,69^. We isolated hippocampal neurons from E18 rat pups and cultured them on a glial monolayer. Neurons were co-transfected at 2 days *in vitro* (DIV) with a plasmid expressing GFP to visualize neuronal morphology, and either a plasmid expressing the human TDP-43 gene under the control of a strong promoter (pCMV) at a concentration of 200 ng/well or 500 ng/well in a 24-well plate, or an empty vector control at 500 ng/well (Fig. 1a). At DIV 7, the cells were fixed and imaged, and dendritic complexity was quantified by Sholl Analysis^70^. Using this approach, we found that TDP-43 overexpression led to reduced dendritic branching (Fig. 1a,b). Increasing the concentration of the TDP-43-expressing plasmid during transfection further decreased the complexity of the dendritic arbor (Fig. 1a,b), indicating that TDP-43-mediated branching defects are dose-dependent.

As the cortex is primarily affected in FTD, we next sought to determine the effect of TDP-43 overexpression on dendritic arborization of dissociated cortical neurons from rodents. Similar to our result using hippocampal neurons, TDP-43 overexpression caused reduced dendritic branching in cultured cortical neurons in a dose-dependent manner (Fig. 1c,d). We traced individual neurites and found that TDP-43 overexpressing neurons also exhibit decreased total length for primary, secondary and tertiary neurites (Supplementary Fig. S1a). Further, neurons overexpressing TDP-43 display fewer secondary and tertiary branches (Supplementary Fig. S1b). By contrast, we found that control neurons and neurons overexpressing TDP-43 have the same number of primary neurites (Supplementary Fig. S1b), perhaps because these neurites formed prior to the time of transfection and TDP-43 overexpression^71–74^. Taken together, these data indicate that overexpression of TDP-43 reduces dendritic complexity in neurons isolated from two different brain regions that are also affected in human patients with ALS or FTD: hippocampus and cortex.

We were interested in understanding whether reduced dendritic branching was a general phenotype associated with overexpression of other ALS/FTD related genes. FUS (Fused in Sarcoma, also known as TLS) is an RNA-binding protein that is genetically linked to ALS and FTD and is structurally and functionally similar to TDP-43^75^; FUS is also ubiquitously expressed and predominantly localized to the nucleus, like TDP-43^76^. We tested the effect of FUS overexpression on dendritic morphology of cortical neurons using the same vector utilized previously for TDP-43 overexpression (pCMV). Neurons were co-transfected with GFP and either an empty vector control (500 ng/well), TDP-43 (500 ng/well), or FUS (500 ng/well). We analyzed dendritic complexity of the transfected neurons using Sholl analysis and found that FUS overexpression did not significantly alter dendritic branching compared to controls (Supplementary Fig. S2), indicating that decreased dendritic arborization is specific to manipulation of TDP-43.

To determine if reduced dendritic complexity could be attributed to poor cell health induced by TDP-43 overexpression, we asked if cortical neurons overexpressing TDP-43 displayed increased cell death relative to control transfected neurons. We assayed cell death using propidium iodide (PI), a membrane-impermeable nucleic acid stain that labels nuclei of dead cells. We quantified the percentage of transfected cells (co-labeled with GFP) that were positive for PI at DIV 4 or DIV 7 in control or TDP-43 overexpressing neurons that were transfected at DIV 2. We found no significant difference in the percentage of PI-positive neurons between the two conditions (Supplementary Fig. S3), indicating that reduced dendritic branching in neurons overexpressing TDP-43 is not a result of enhanced cell-death within this time-frame.

TDP-43 has been shown to regulate the expression of microtubule stabilizing proteins, such as MAP1B at *Drosophila* neuromuscular junctions^45,46^. Given that microtubule stabilization plays a major role in establishing and maintaining dendritic architecture, we asked whether there are similar changes in the expression of another microtubule regulator specifically found in neurons, Microtubule Associated Protein-2 (MAP2), which promotes microtubule stability in dendrites. We measured the levels of MAP2 in cortical neurons after overexpression of TDP-43 at 500 ng/well (Supplementary Fig. S4a). MAP2 protein levels in dendrites did not change significantly in the TDP-43 overexpression condition (Supplementary Fig. S4b), indicating that the reduction in dendritic branching does not result from a loss of MAP2 expression.

### TDP-43 knockdown leads to reduced dendritic complexity

**Figure 2:**
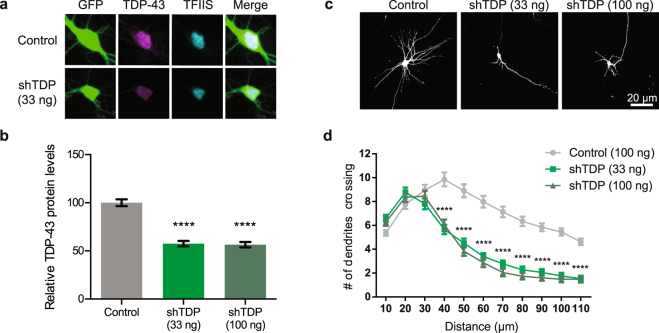
Knocking down TDP-43 expression results in reduced dendritic complexity. (**a**) Representative images of DIV 7 cortical neurons transfected on DIV 2 with GFP and either an empty vector or a short-hairpin targeting TDP-43 (shTDP; 33 ng/well). Neurons were stained using antibodies recognizing TDP-43 (magenta) and TFIIS (cyan) as a control. (**b**) Average fluorescence intensity of TDP-43 immunostaining in the soma (nucleus and cytoplasm) normalized to average fluorescence intensity of TFIIS in the nucleus (Control, N = 65; shTDP (33 ng/well), N = 58; shTDP (100 ng/well), N = 48; One-Way ANOVA with Tukey’s test, ****p < 0.0001. **(c)** Representative images showing DIV 7 cortical neurons transfected on DIV 2 with shRNA targeting TDP-43 (shTDP) at either 33ng/well or 100 ng/well or an empty vector control at 100 ng/well. (**d**) Sholl analysis comparing dendritic arbors of control neurons and shTDP neurons at the indicated concentrations (Control, N = 64; shTDP (33 ng/well), N = 50; shTDP (100 ng/well), N = 49). Two-Way ANOVA with Tukey’s test ****p < 0.0001. *represents comparisons between both shTDP conditions and the control condition.

Given that depletion of nuclear TDP-43 is observed in ALS and FTD patient tissue^8^ and that TDP-43 LOF causes ALS-like phenotypes in mice^77–79^, we investigated whether TDP-43 knockdown affected neuronal morphology. We transiently transfected cortical neurons with GFP and a vector expressing previously validated shRNAs against TDP-43^67^ (33 ng/well or 100 ng/well), or an empty vector control. On DIV 7, we fixed these neurons, and confirmed depletion of TDP-43 by immunostaining with a polyclonal antibody recognizing TDP-43 (Fig. 2a). Relative levels of TDP-43 expression in both the nucleus and cytoplasm were calculated by normalizing intensity of TDP-43 staining within the soma to intensity of staining with an antibody that recognizes TFIIS, a ubiquitously expressed transcription elongation factor that is localized to the nucleus^80^ and is not expected to be affected by changes in TDP-43 expression levels (Fig. 2b). We then quantified neuronal morphology by Sholl analysis, and found that TDP-43 depletion, similar to TDP-43 overexpression, resulted in reduced dendritic complexity, consistent with previous reports^67^ (Fig. 2c,d). Both concentrations of the hairpin reduced the level of TDP-43 protein to a similar extent, indicating that increasing the dosage of hairpin doesn’t result in further reduction of TDP-43 protein or increased severity of the dendritic branching phenotype. Thus, both GOF and LOF of TDP-43 result in similar dendrite complexity phenotypes, suggesting that both decreased and increased TDP-43 expression causes misregulation of TDP-43 targets that control dendritic elaboration.

### Dendritic growth is suppressed by TDP-43 overexpression

**Figure 3:**
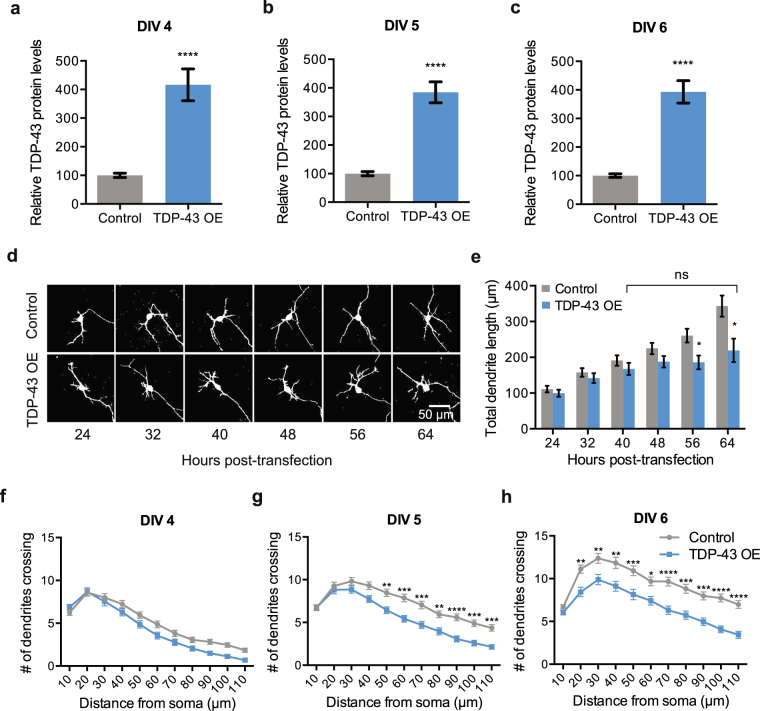
TDP-43 overexpression reduces dendritic complexity by suppressing dendritic growth. (**a**) Average fluorescence intensity of TDP-43 immunostaining in the soma (nucleus and cytoplasm) normalized to average fluorescence intensity of TFIIS in the nucleus at DIV 4 (Control, N = 59, TDP-43 overexpression, N = 61), (**b**) DIV 5 (Control, N = 59, TDP-43 overexpression, N = 60), and **(c)** DIV 6 (Control, N = 60, TDP-43 overexpression, N = 60), respectively. ****p < 0.0001, unpaired t-test. (**d**) Representative images of an individual cortical neuron transfected with either an empty vector (control) or 500 ng/well TDP-43 (TDP-43 OE), repeatedly imaged at 8 hour intervals as indicated. (**e**) Average total dendrite length for each condition plotted as a function of time. *p < 0.05 for the comparison between TDP-43 overexpression and control condition using Unpaired *t*-test. Average total dendrite length for TDP-43 overexpression condition is not significantly different between 40 and 64 hours (Repeated Measures Two-Way ANOVA with Sidak’s multiple comparison). Control, N = 54; TDP-43 overexpression, N = 24. (**f–h**) Sholl analysis of hippocampal neurons comparing dendritic arbors between control and TDP-43 overexpressing neurons transfected on DIV 2 and fixed on (**f**) DIV 4 (Control, N = 52; TDP-43 overexpression, N = 54), (**g**) DIV 5 (Control, N = 62; TDP-43 overexpression, N = 61), and (**h**) DIV 6 (Control, N = 61; TDP-43 overexpression, N = 60). Two-way ANOVA with multiple comparisons, *p < 0.05, **p < 0.01, ***p < 0.001, ****p < 0.0001.

Elaboration of the dendritic arbor is a dynamic process involving both growth and retraction of extending neurites^81^. Although TDP-43 overexpressing neurons display reduced dendritic branching, it remains unclear how this phenotype arises. We first quantified the expression levels of TDP-43 protein in neurons transfected with either an empty vector (500 ng/well) or TDP-43 (500 ng/well) over the time-course of our experiment, using a polyclonal TDP-43 antibody that recognizes both rat and human TDP-43, starting at 2 days post-transfection. Relative levels of TDP-43 expression were calculated by normalizing intensity of TDP-43 staining within the nucleus and cytoplasm to intensity of staining with a control antibody labeling TFIIS^80^. At DIV 4 (2 days post-transfection), we observed that neurons transfected with the TDP-43-expressing plasmid displayed approximately a 4-fold increase in TDP-43 protein levels compared to controls (Fig. 3a); this increased TDP-43 protein level remained constant for the remainder of the experiment (Fig. 3b,c). Given that TDP-43 predominantly localizes to the nucleus, we were curious whether TDP-43 protein levels were elevated in a specific subcellular compartment. To answer this question, we measured average fluorescence intensity for TDP-43 immunostaining within the Hoechst-labeled nuclear area or the GFP-filled cytoplasm in control and TDP-43 overexpressing neurons (Supplementary Fig. S5). We found that TDP-43 protein levels were increased in both the nucleus (Supplementary Fig. S5b) and the cytoplasm (Supplementary Fig. S5c). In addition, since TDP-43 has been reported to form pathological protein inclusions in post-mortem brain tissue, we asked if TDP-43 formed protein aggregates in our cell culture system by TDP-43 immunostaining. We did not observe TDP-43 inclusion formation by DIV 6 in our system. Instead, TDP-43 overexpression resulted in a diffuse localization pattern in both the nucleus and cytoplasm (Supplementary Fig. S5a).

We reasoned that a TDP-43-dependent reduction in dendritic branching could be due to either a suppression of dendrite growth, or an increase in dendrite retraction. We used repeated imaging of individual cortical neurons over a period of 40 hours to track the effect of TDP-43 overexpression on dendrite extension. Cortical neurons were plated on separate 35 mm dishes and co-transfected 4 hours after plating with a plasmid expressing TdTomato to visualize neuronal morphology and either an empty vector (2 µg/dish) or a TDP-43-expressing plasmid (2 µg/dish). These vector concentrations scale proportionally with our previous transfections on 24-well plates at 500ng/well. The dendrites from each image were traced and measured for total dendrite length at each time point. We observed that dendrites from control neurons increased in length at each time point over the 40-hour imaging window (Fig. 3d,e). However, TDP-43-overexpressing neurons exhibited a reduced rate of dendritic growth compared to control neurons, and by 40 hours post-transfection halted their growth altogether (Fig. 3d,e), suggesting that TDP-43 overexpression suppresses dendritic outgrowth.

Similarly, hippocampal neurons overexpressing TDP-43 showed a consistent reduction in neurite outgrowth by Sholl analysis over time. We transfected hippocampal neurons with either a control plasmid (500 ng/well) or a TDP-43 plasmid (500 ng/well) and then fixed and imaged a subset of these neurons on each successive day starting at 2 days post-transfection (DIV4). We found that TDP-43 overexpressing neurons did not exhibit a statistically significant difference in dendritic complexity at DIV 4 (48 hours after transfection) (Fig. 3f). However, while the control transfected neurons increased their dendritic complexity from DIV4–DIV 6 by approximately 2-fold (e.g. the 50 µm circle radius), the TDP-43-overexpressing dendritic arbors did not change as drastically (Fig. 3g-h). Taken together, we conclude from these two experiments that decreased dendritic arborization in the TDP-43 overexpression condition is due to a suppression of dendritic growth starting approximately 40 hours after transfection which persists for at least 48 more hours.

### Human TDP-43 mutations do not exacerbate the reduced branching phenotype

**Figure 4:**
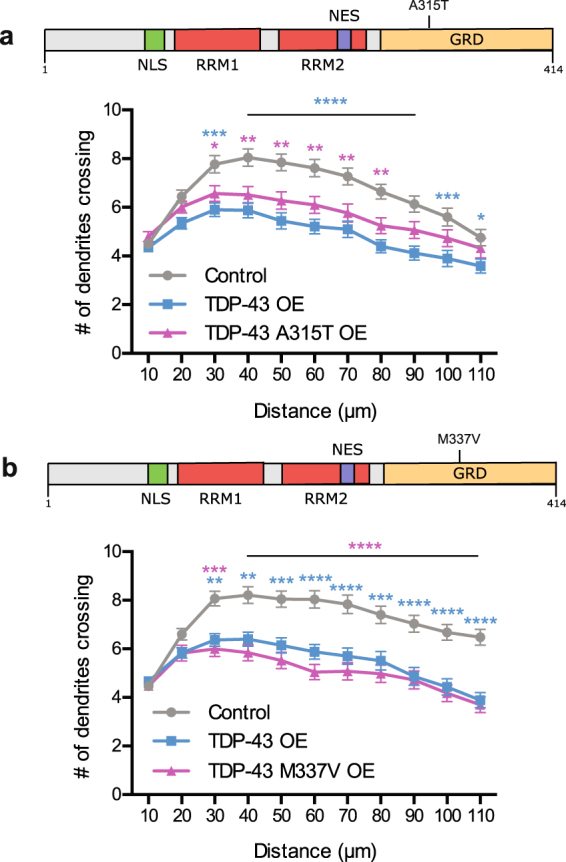
Human TDP-43 mutations do not exacerbate the reduced branching phenotype (**a**) (top) Schematic of TDP-43 protein demonstrating location of A315T point mutation. NLS: Nuclear Localization Signal, RRM1, RRM2: RNA Recognition Motif, NES: Nuclear Export Signal, GRD: Glycine Rich Domain. (bottom) Sholl analysis for indicated conditions (Control, N = 60, TDP-43 overexpression, N = 60, TDP-43 A315T overexpression, N = 60). (**b**) (top) Schematic of TDP-43 protein demonstrating location of M337V point mutation; abbreviations as in a. (bottom) Sholl analysis for indicated conditions (Control, N = 60, TDP-43 overexpression, N = 60, TDP-43 M337V overexpression, N = 50). Two-Way ANOVA with Tukey’s test, *p < 0.05, **p < 0.01, ***p < 0.001, ****p < 0.0001. The colour of the *represents the experimental condition being tested against control.

To date, over 50 nonsynonymous mutations in the human TDP-43 gene (*TARDBP*) have been identified in both familial and sporadic ALS patients^82,83^. Most of these mutations reside in the C-terminal glycine rich domain, which is essential in mediating protein-protein interactions^49^. A few of these mutations have been shown to impair the trafficking of TDP-43-containing RNP granules in axons^41^. In addition, mutant forms of TDP-43 have been reported to increase TDP-43 cytoplasmic localization in cultured cortical neurons^68^. Thus, we hypothesized that TDP-43 mutations might exacerbate the decreased dendritic branching phenotype observed upon overexpression of wild-type (WT) TDP-43. To test this hypothesis, we generated two TDP-43 constructs, each carrying a single point mutation previously described in human patients: TDP-43 A315T and TDP-43 M337V (Fig. 4a,b). We tested the effect of overexpressing these mutant forms of TDP-43 on dendritic branching by Sholl analysis at DIV 7, following 5 days of TDP-43 WT or mutant transfection. Interestingly, overexpression of either TDP-43 mutant caused a reduction in dendritic branching similar to the overexpression of WT TDP-43 (Fig. 4a,b). Our finding that the M337V and the A315T mutations do not cause a more severe dendritic growth phenotype than WT TDP-43 suggests that the TDP-43 dependent-signaling pathways which regulate dendritic branching are not additionally affected by these mutations.

### Inhibition of dendritic branching by TDP-43 overexpression depends on TDP-43 N-terminus and RNA-binding

**Figure 5:**
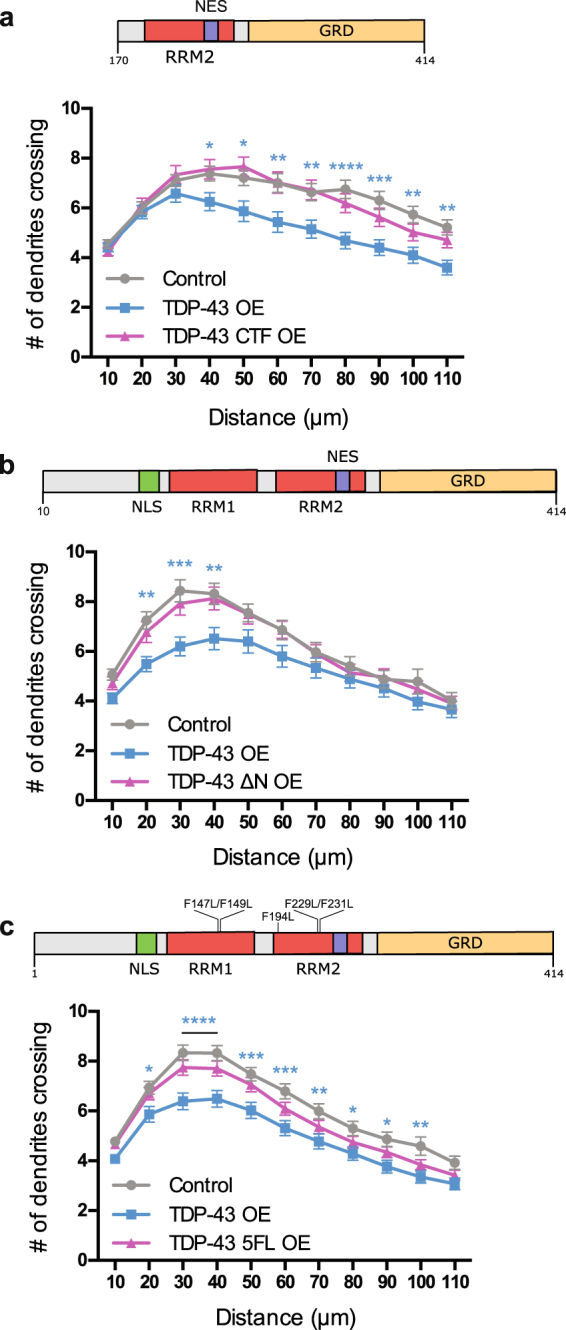
RNA binding ability of TDP-43 is important for regulating dendritic branching (**a**) (top) Schematic of TDP-43 protein demonstrating the C-terminal fragment (CTF, amino acid residues 170–414) being overexpressed. RRM2: RNA Recognition Motif, NES: Nuclear Export Signal, GRD: Glycine Rich Domain. (bottom) Sholl analysis for indicated conditions (N = 60 for all conditions). The colour of the *represents the experimental condition being tested against control. (**b**) (top) Schematic of TDP-43 protein demonstrating TDP-43 ∆N (amino acid residues 10–414). NLS: Nuclear Localization Signal, RRM1: RNA Recognition Motif. (bottom) Sholl analysis for indicated conditions (Control, N = 57, TDP-43 overexpression, N = 45, TDP-43 ∆N overexpression, N = 54). (**c**) (top) Schematic of TDP-43 protein demonstrating TDP-43 5FL (F147L/F149L/F194L/F229L/F231L). (bottom) Sholl analysis for indicated conditions (Control, N = 87, TDP-43 overexpression, N = 76, TDP-43 5FL overexpression, N = 90). The colour of the *represent the experimental condition being tested against control. (**a–c**) Grey vs pink = not significant. Two-Way ANOVA with Tukey’s test, *p < 0.05, **p < 0.01, ***p < 0.001, ****p < 0.0001.

Initial reports from the literature indicate that protein inclusions found in cortical neurons are predominantly composed of TDP-43 C-terminal fragments (CTFs)^7,8,84^. Numerous reports have also indicated that these CTFs are toxic to neurons^85–90^. For example, overexpression of CTFs in cultured rat forebrain neural precursors impairs neurite formation during neuronal differentiation^90^. TDP-43 CTF lacks its native nuclear localization signal (NLS)^33^ and it has been shown to be enriched in the cytoplasmic compartment^90^. Furthermore, TDP-43 CTF also lacks its primary RNA-recognition motif (RRM1), so it cannot interact with its RNA targets^26^. We sought to determine if overexpression of the C-terminal domain could also suppress dendritic branching. To address this question, we co-transfected cultured cortical neurons on DIV 2 with a GFP-expressing plasmid along with an empty vector (500 ng/well), or TDP-43 (500 ng/well), or a plasmid expressing a truncated TDP-43 (500 ng/well) which only expresses the C-terminal region of TDP-43 composed of amino acid residues 170–414 (hereafter called TDP-43 CTF) (Fig. 5a). These neurons were fixed on DIV 7 and assayed for dendritic complexity by Sholl analysis. We found that overexpression of TDP-43-CTF had no effect on dendritic complexity (Fig. 5a). These results indicate that the C-terminal domain of TDP-43, consisting of residues 170–414, is not sufficient to suppress dendritic growth, suggesting that perhaps the N-terminus of TDP-43 is required for this function.

Recently, the N-terminal domain of TDP-43 has received much attention. Several groups have demonstrated that the N-terminus of TDP-43 contributes to the formation of homodimers^91–94^ and enhances splicing activity^93,94^. Therefore, we investigated whether the overexpression of a TDP-43 construct containing amino acid residues 10–414 (hereafter referred to as TDP-43 ∆N) was sufficient to suppress dendritic branching. Cultured cortical neurons were co-transfected on DIV 2 with a GFP-expressing plasmid along with an empty vector (500 ng/well), or TDP-43 (500 ng/well), or TDP-43 ∆N (500 ng/well). On DIV 7, these neurons were fixed and assayed by Sholl analysis. We found that TDP-43 ∆N overexpression had no effect on dendritic branching (Fig. 5b), suggesting that homodimerization and/or splicing function may play a role in mediating reduced dendritic complexity.

TDP-43 is a RNA binding protein involved in several aspects of RNA metabolism including splicing, transport, and translation^41,45,95^. One model to explain our observations is that TDP-43 GOF or LOF causes misregulation of one or more of its target mRNAs, which regulate dendritic growth. Therefore, we asked if overexpression of a mutant form of TDP-43 that cannot bind RNA results in reduced dendritic complexity. We addressed this question by introducing five point mutations into TDP-43, F147/149 L in RRM1 and F194/229/231 L in RRM2, that have been shown to abolish its RNA-binding ability (hereafter called TDP-43 5FL)^26^. We co-transfected cortical neurons with a GFP-expressing plasmid along with an empty vector (500 ng/well), TDP-43 (500 ng/well), or a plasmid expressing TDP-43 5FL (500 ng/well) on DIV 2 and fixed these neurons on DIV 7. Overexpression of TDP-43 5FL had no effect on dendritic complexity (Fig. 5c), suggesting that the ability of TDP-43 to inhibit dendritic branching is due to its RNA-binding function.

## Discussion

In this study, we demonstrate that increasing levels of TDP-43 in cultured mammalian neurons suppresses dendritic growth. This is in contrast to overexpression of the RNA binding protein and ALS gene, FUS, which had no effect on dendrite growth. Abnormal dendritic morphology is a common characteristic of several different neurological disorders^96–103^ and reduced dendritic complexity has been reported to precede neurodegeneration^104^. Importantly, TDP-43 overexpression also causes reduced dendritic complexity in both hippocampal and cortical neurons. The hippocampus and cortex are two regions of the brain that are prone to neurodegeneration in both ALS and FTD^105^. Cell morphological changes such as reduced dendritic complexity could interfere with circuit connectivity and disrupt cell to cell communication, ultimately affecting cognition and motor function.

In addition, we and others^67^ have demonstrated that RNAi-mediated TDP-43 knockdown in cortical neurons results in reduced dendritic branching. Indeed, TDP-43 knockdown and overexpression in several systems has resulted in similar cell morphological and behavioral phenotypes^15–17^. For example, both GOF or LOF of TDP-43 cause motor deficits in zebrafish^15^, defects in growth receptor signaling^16^, and affect expression of a set of proteins in a similar manner^17^. Taken together, these results suggest that both increased or decreased levels of TDP-43 negatively affects neuron function, perhaps via misregulation of a subset of critical TDP-43 targets^13,106^. Alternatively, TDP-43 LOF and GOF could affect dendritic growth through distinct mechanisms or targets.

We also asked if TDP-43 overexpression affected dendritic arborization by suppressing dendrite growth or promoting dendrite retraction. We distinguished between these mechanisms by performing a time-lapse analysis of dendrite elaboration in living cortical neurons overexpressing TDP-43, and an examination of neurite growth and branching in fixed neurons transfected with TDP-43 at 2 days, 3 days, and 4 days post-transfection. We found that dendritic growth is suppressed when TDP-43 protein levels are elevated, consistent with our live-imaging data in which TDP-43 overexpressing neurons stop dendritic growth at approximately 40 hours post-transfection. Thus, increased levels of TDP-43 alter dendritic complexity by suppressing dendritic growth, providing insight into possible cellular mechanisms by which TDP-43 regulates dendritic arborization.

Given that TDP-43 is an RNA-binding protein that regulates the splicing, transport, and stability of thousands of RNA transcripts, we asked if the RNA binding ability of TDP-43 was required to suppress dendritic branching. Interestingly, our data shows that reduction in dendritic branching due to TDP-43 overexpression is dependent on this function. This result is reminiscent of studies in *C*. *elegans*, *D*. *melanogaster*, and *G*. *gallus* models in which phenotypes associated with TDP-43 overexpression are abolished by disrupting TDP-43 RNA-binding ability^107–109^. Specifically, overexpression of wild-type TDP-43 led to a reduction of motor neuron number in *G*. *gallus* spinal cord while expression of an RNA-binding deficient TDP-43 did not cause motor neuron loss^107^. Thus, our results suggest that TDP-43 overexpression interferes with one or more RNA binding functions that is relevant to dendritic growth and branching.

At least one important function of TDP-43 that requires RNA-binding is splicing. Interestingly, the N-terminus of TDP-43 has been reported to play a vital role in splicing activity^93,94^. Therefore, we investigated whether overexpressing a TDP-43 construct lacking the first 9 amino acid residues could affect dendritic complexity. Consistent with previous reports, the extreme N-terminus of TDP-43 is required to suppress dendritic outgrowth^93^. TDP-43 ∆N still contains both RRMs and is presumably still capable of an RNA-binding interaction. Thus, our results indicate that, after RNA-binding, the splicing function of TDP-43 may also be necessary to suppress dendritic elaboration. However, we cannot rule out other possible functions that may become defective upon truncation of the TDP-43 N-terminus that lie downstream of RNA-binding such as RNA transport or translation repression.

Similarly, CTFs are a pathological hallmark of ALS and FTD^8,84^, are enriched in the cytoplasm^90^ due to loss of the NLS, and are prone to aggregation^88^. It is hypothesized that CTFs may act as a sink and recruit wild-type TDP-43 to form pathological protein inclusions^90,110–112^. While a previous study demonstrated that overexpression of CTFs impairs neurite growth in NSC-34 cells^90^, we observed that overexpression of TDP-43 CTFs in mammalian cortical neurons does not alter dendrite morphology. The disparity in results between our two studies may lay in the fact that different cell types were employed for these experiments. Our results indicate that the pathological function of CTFs is independent of an effect on dendrite elaboration. Importantly, because the CTF lacks the TDP-43 RNA binding domain, these results support our conclusion that RNA binding ability is required for TDP-43 inhibition of dendrite growth.

In conclusion, our data indicate that either increasing or decreasing TDP-43 levels has negative consequences for dendrite elaboration in both cortical and hippocampal neurons, which could contribute to the pathology of diseases such as ALS and FTD. Although the molecular mechanisms behind TDP-43 mediated regulation of dendritic architecture remain to be elucidated, our data demonstrate that TDP-43 RNA-binding function plays a major role in altering neuronal morphology. In addition, our results further support the relevance of studying TDP-43 overexpression in neuronal cultures, as the phenotypic consequences of this genetic manipulation is dependent upon a well-established biological function of TDP-43. Thus, a careful examination of RNA misregulation in our culture system could yield insights into signaling pathways that are perturbed upon TDP-43 misexpression and are relevant to dendritic growth. This system can potentially provide a platform to test avenues for therapeutic intervention in ALS/FTD.

## Methods

The datasets generated during and/or analyzed during the current study are available from the corresponding author on reasonable request. All animal procedures were approved by the Brandeis University Institutional Animal Care and Usage Committee and all experiments were performed in accordance with relevant guidelines and regulations.

### Plasmids

Plasmids expressing full length human TDP-43 (Addgene plasmid # 28206) and C-terminal truncated human TDP-43 (hTDP-43^170–414^; Addgene plasmid # 28196) were a gift from Dr. Zuoshang Xu (University of Massachusetts Medical School, Worcester MA)^90^. Plasmids expressing full-length human FUS (Addgene plasmid # 29609) and TDP-43 5FL (Addgene plasmid # 84914) was a gift from Aaron Gitler (Stanford University School of Medicine, Stanford CA). pCSCMV:tdTomato was a gift from Gerhart Ryffel (Addgene plasmid # 30530). Full-length TDP-43, FUS, and TDP-43 5FL were cloned into pCMV-myc vector backbone using Gibson Assembly (NEB). Constructs containing various mutations of TDP-43 were generated from pCMV-myc hTDP-43 plasmid by site-directed mutagenesis using QuickChange Site Directed Mutagenesis Kit (Stratagene, San Diego CA). TDP-43 ∆N was generated by cloning from the pCMV-myc hTDP-43 plasmid. Primers used to generate indicated mutations are as follows:

M337V: GAGCAGTTGGGGTATGGTGGGCATGTTAGCC

A315T: TGGTGGGATGAACTTTGGTACGTTCAGCATTAATCC

∆N: GAGGCCCGAATTCGGGGACGATGGTGTGACTGC

### Primary Neuronal Cultures and Transfection

Dissociated rat cortical or hippocampal neurons were cultured on a feeder layer of astrocytes as described previously^113^. Briefly, a layer of confluent astrocytes was generated by plating the cells onto 12 mm glass coverslips coated with poly-D-lysine (20 μg/ml) and laminin (3.4 μg/ml) in 24 well plates. Dissociated hippocampal or cortical neurons from E18 rat embryos were plated on this layer of astrocytes at the density of 80,000 cells/well, and grown in Neurobasal medium supplemented with B27 (Thermo Fisher) at 37 °C.

Neurons were transfected at the desired stage by using the calcium phosphate transfection method^114^, which yields a low transfection efficiency, allowing development of transfected neurons within a large population of unaffected neurons, as well as better visualization of neuronal morphology for individual transfected neurons. All experimental conditions were co-transfected with pCMV-GFP plasmid at 500 ng/well to visualize cellular morphology. In addition, all wells were co-transfected with either pCMV-myc plasmid for control empty-vector conditions, or other plasmid DNA as described.

### Analysis of Neuronal Morphology

Transfected neurons were fixed at the desired time-point using 4% paraformaldehyde + 4% sucrose solution in PBS for 8 minutes at room temperature followed by 3 washes in 1X PBS. Images of individual transfected neurons were acquired on Olympus Fluoview 300 confocal microscope using a 20X oil objective (numerical aperture [NA] 0.85) in order to image the entire dendritic arbor (5–10 steps at 1 μm optical sectioning). All conditions were imaged at similar acquisition settings for laser power and detector gain in a blinded manner, and unblinded only after analysis.

### Image Analysis

Maximum Intensity Projections (MIP) were generated from individual Z stacks using ImageJ. For Sholl analysis, a series of 11 concentric circles (10 μm intervals) centered at the soma, starting at 10 µm, was overlaid on the MIP using the ‘Concentric circles’ plugin. The number of dendrites crossing each circle was measured manually. For each experimental condition, a total of 15–30 neurons were analyzed from 2–3 coverslips with 5–15 neurons per covers lip. The reported results combine data from at least 2 independent biological replicates. For measuring total neurite length and branch tip number, neurites were traced using the NeuronJ plugin in ImageJ. The sum length of all the neurites was calculated for 10–15 neurons from 2–3 coverslips and the results were combined from 2 independent biological replicates.

To measure TDP-43 protein levels, sum projections were generated from individual Z stacks with ImageJ. In ImageJ, using GFP as a marker for transfected cells, a region of interest was drawn around the soma and fluorescence intensity was measured in that area in the TDP-43 channel. TFIIS fluorescence intensity was measured by drawing a region of interest around the Hoechst-33342-labeled nucleus, and fluorescence intensity was measured in the TFIIS channel. TDP-43 intensity was normalized to TFIIS intensity for every cell.

Nuclear vs cytoplasmic levels of TDP-43 staining were measured as follows. For nuclear TDP-43 levels, the region of interest was defined by the Hoechst-33342 labeling and TDP-43 intensity was measured in the TDP-43 immunostained channel. The TDP-43 levels in the cytoplasm were determined by drawing a region of interest around the GFP-filled soma, measuring TDP-43 intensity in the TDP-43 channel, and subtracting the previously measured nuclear TDP-43 intensity.

MAP2 levels were quantified by measuring fluorescence intensity of MAP2 immunostaining from empty vector or TDP-43-transfected cells (500 ng/well). Sum projections of each condition were generated using ImageJ, with GFP as a marker for transfected cells. Average intensity was calculated by taking the mean intensity from 2 separate dendrites of a transfected neuron followed by normalizing to the average intensity from 2 other dendrites from non-transfected cells.

### Cell death Assay

Hippocampal neurons were cultured in 24-well plates as described and transfected with pCMV-GFP plasmid along with either empty pCMV-myc control plasmid at 500ng/well or full-length hTDP-43 at 500 ng/well. Neurons were incubated in 2 µg/ml Propidium Iodide solution (Life Technology) in HEPES buffer (117 mM NaCl, 5.3 mM KCl, 1.8 mM CaCl_2_, 0.814 mM MgSO_4_, 1 mM NaH_2_PO_4_, 20 mM HEPES, 50 mM D-glucose, 0.1% BSA, 320 mOsm, pH 7.2) for 1 hour at 37 °C followed by 1X PBS wash. Cultures were fixed with 4% PFA + 4% sucrose solution in PBS. GFP-positive neurons were counted manually using a 60x objective and an epiflourescence microscope, and the presence or absence of PI fluorescence was noted for each GFP-positive neuron by flipping between filter sets. For each experimental condition, 2 coverslips each from 2 independent experiments were analyzed.

### Immunostaining

Following primary antibodies were used for immunostaining, α-TDP-43 (10782-2-AP) from ProteinTech (used at 1:500); α-TFIIS (611204) from BD Transduction Laboratories (used at 1:275); α-MAP2 (M4403) from Sigma-Aldrich (used at 1:1000). Secondary antibodies were conjugated to Cy-3 or Cy-5 (1:500, Jackson ImmunoResearch Laboratories). Coverslips containing transfected neurons were fixed as described above followed by 3 washes with 1X PBS. The coverslips were then incubated with primary antibodies) diluted in gelatin blocking buffer at 4 °C overnight in a humidified chamber (30 minutes at room temperature for MAP2). Coverslips were then washed 3 times with 1X PBS and incubated with secondary antibodies at room temperature for 2 hours. Coverslips were then washed 3 times with 1X PBS, submerged in MilliQ water and then mounted on glass microscope slides with Aquamount (Lerner Laboratories). Neurons were imaged using an Ni-E inverted microscope equipped with a Nikon C2 confocal head and a 60X oil (NA 1.4) objective, using similar acquisition settings for laser power, offset and detector gain across conditions in a blinded manner.

### Live Imaging

An astrocyte feeder layer and cortical neurons were plated as described above on 35 mm glass-bottom dishes. 4 hours after plating, neurons were transfected via the calcium phosphate method with a plasmid expressing TdTomato to fill the cell and an empty vector control or a vector expressing TDP-43; our empirical observation was that TdTomato is less phototoxic than GFP for these live imaging experiments. The next day (~24 hours later) the neurons were placed in an OKO labs temperature controlled environmental chamber (5% CO2, 95% O2) and 20–30 neurons were randomly selected from each condition. Images of the selected neurons were acquired every 8 hours for 40 hours using an Ni-E inverted microscope equipped with a Nikon C2 confocal head and a 40X (NA 0.95) objective. Images were collected using Nikon Elements AR software. The experimenters were not blinded to the condition during the selection of neurons but were blinded to each condition during analysis. Images from each time point were analyzed by tracing individual dendrites using ImageJ.

## Electronic supplementary material

Supplementary Information
