# Supplementary material for: TDP-43 misexpression causes defects in dendritic growth
Source: Sci Rep. 2017 Nov 15;7:15656. doi: 10.1038/s41598-017-15914-4 (PMC5688077; doi:10.1038/s41598-017-15914-4)

TDP-43 misexpression causes defects in dendritic growth

Josiah J. Herzog[1], Mugdha Deshpande[1], Leah Shapiro, Avital A. Rodal, Suzanne Paradis*

[1] These authors contributed equally

Supplementary Information:

Figure S1: TDP-43 overexpression affects neurite branching and length

(a) Average total length and **(b)** average branch tip number for primary, secondary, and tertiary neurites of DIV 7 cortical neurons transfected on DIV 2 with GFP and either an empty vector (control) or TDP-43 (Control, N=51; TDP-43 overexpression, N=46). Unpaired t-test, * p <0.05, ** p<0.01, *** p <0.001, **** p< 0.0001.

Figure S2: Overexpression of FUS has no effect on dendritic architecture

Sholl analysis for DIV 7 cortical neurons transfected on DIV 2 with 500 ng/well of either empty vector (Control, N= 62), TDP-43 overexpression (N=74), or FUS overexpression (N=71). Two-Way ANOVA with Tukey's test, * p<0.05, ** p<0.01, *** p <0.001, **** p<0.0001.

Figure S3: TDP-43 overexpression in primary cortical neurons does not exacerbate neuronal cell death

Cortical neurons transfected on DIV 2 with GFP and either an empty vector (500 ng/well) or TDP-43 (500 ng/well) were fixed on **(a)** DIV 4 or **(b)** DIV 7 and stained with propidium iodide (PI). Cell death was quantified as the percentage of PI-positive + GFP-positive neurons divided by total GFP-positive neurons. (DIV 4: Control= 5.56%, TDP-43 overexpression= 8.51%, Chi Square Test P value= 0.38; DIV 7: Control= 4.69%, TDP-43 overexpression= 4.26%, Chi Square Test P value= 0.34).

Figure S4: MAP2 expression levels are unchanged by TDP-43 overexpression

GFP along with either an empty vector (500 ng/well) or TDP-43 (500 ng/well) was transfected into cortical neurons on DIV 2 and these neurons were fixed on DIV 7. **(a)** Representative images for control and TDP-43-overexpressing cortical neurons immunostained with a MAP2 antibody and Hoechst to label the nucleus. White arrows point to nuclei of transfected cells **(b)** Average fluorescence intensity of MAP2 staining from 2 dendrites of transfected cells were normalized to average intensity of MAP2 from 2 separate dendrites from non-transfected cells. (Control n=57 cells, TDP-43 OE n=46 cells). Student's t-test.

Figure S5: TDP-43 overexpression results in higher protein levels in both nucleus and cytoplasm

Quantification of average TDP-43 intensity in cortical neurons transfected at DIV 2 with GFP and either an empty vector or a TDP-43 expressing plasmid and fixed at DIV 4, 5 or 6. **(a)** Representative images for control and TDP-43 overexpressing DIV 6 neurons immunostained with a TDP-43 antibody and Hoechst to mark the nucleus. White arrows point to nuclei of transfected cells. Average TDP-43 intensity was calculated as described for **(b)** nuclear and **(c)** cytoplasmic compartments. DIV 4: Control, N=59; TDP-43 overexpression, N=60; DIV5: Control, N=57; TDP-43 overexpression, N=60; DIV6: Control, N=60; TDP-43 overexpression, N=60. **** $p < 0.0001$, unpaired *t*-Test.

Supplementary Figure S1:

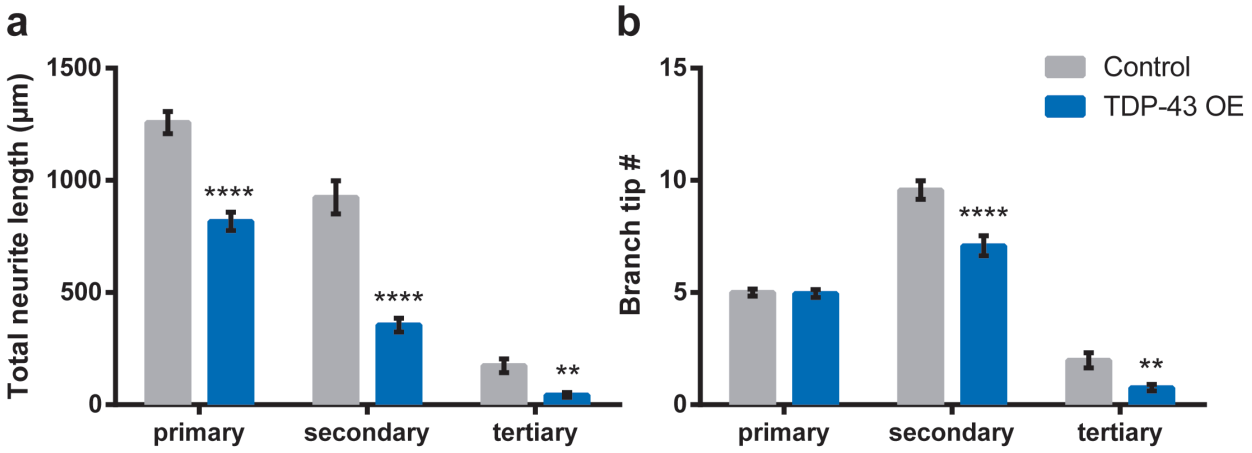

Supplementary Figure S2:

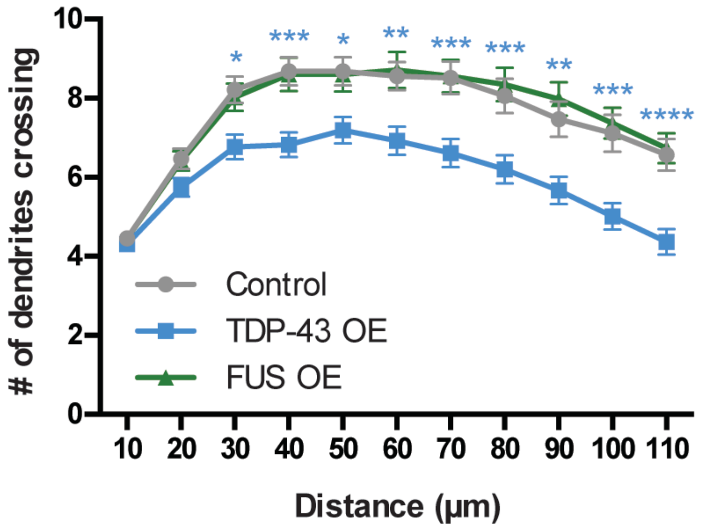

Supplementary Figure S3:

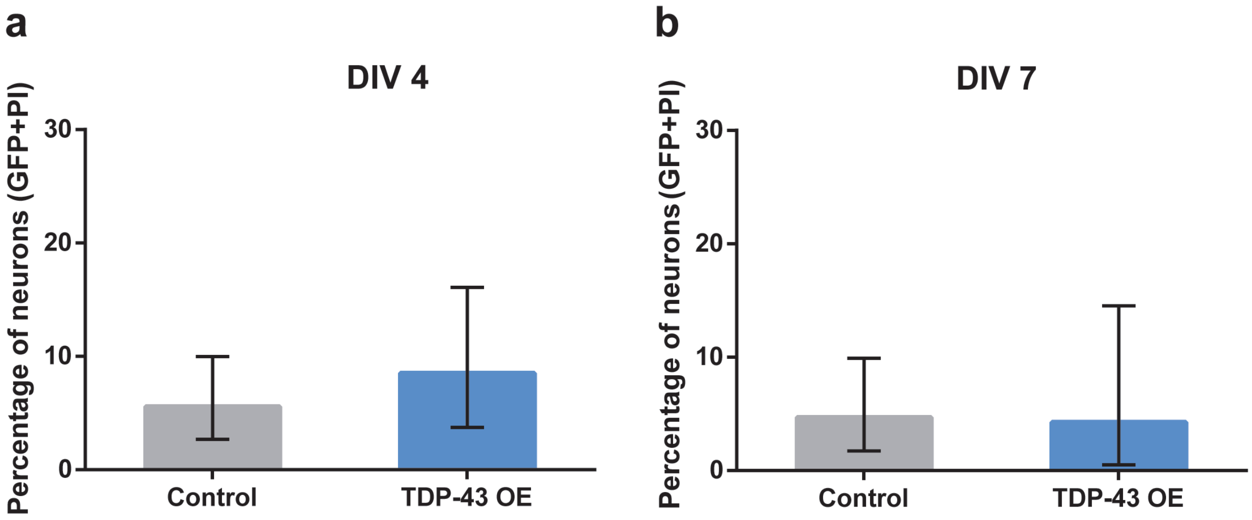

Supplementary Figure S4:

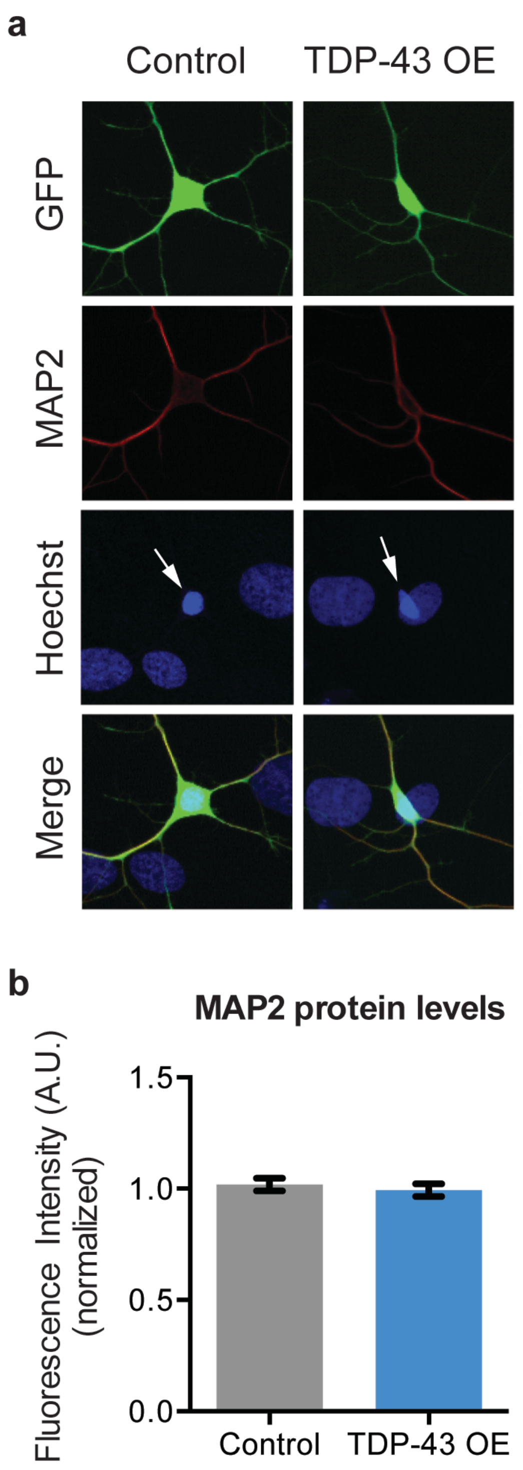

Supplementary Figure S5:

a

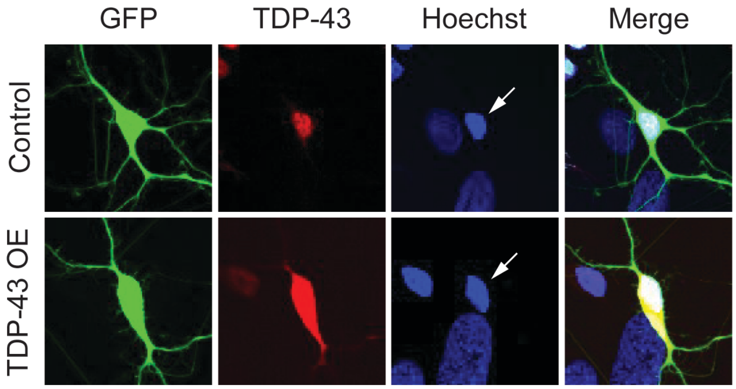

b

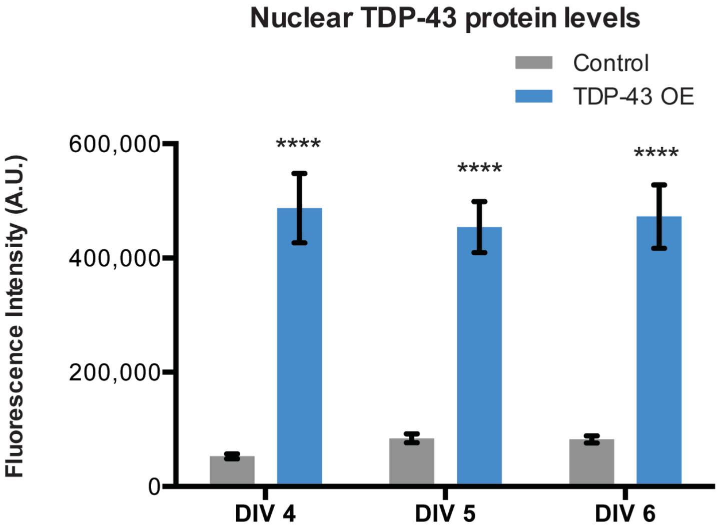

c

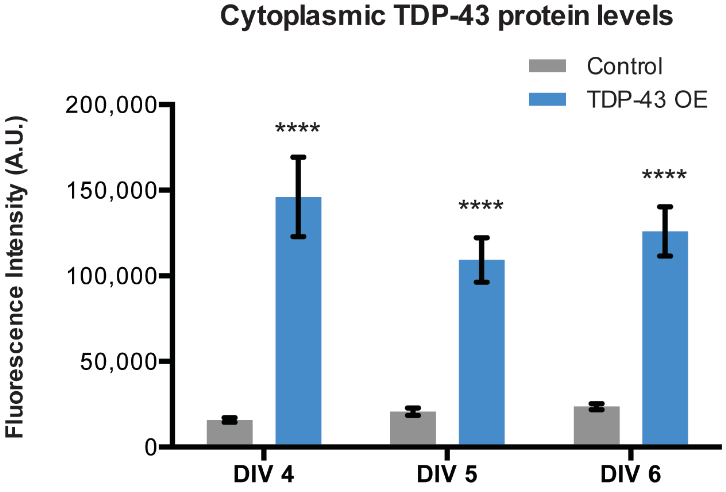

Supplement: Supplementary file 1 — Supplementary Information [file 41598_2017_15914_MOESM1_ESM.pdf]
